# Juvenile idiopathic arthritis and primary ovarian failure: a two-sample Mendelian randomization analysis in a mixed-gender cohort

**DOI:** 10.3389/fendo.2024.1340993

**Published:** 2024-05-16

**Authors:** Yuanhang Mo, Anquan Shang, Guoguo Wei, Donghui Xu, Yuxi Hou, Xiaowen Shao, Chaoyan Yue

**Affiliations:** ^1^ Department of Obstetrics and Gynecology, Shanghai Tenth People’s Hospital, Tongji University School of Medicine, Shanghai, China; ^2^ Department of Laboratory Medicine, The Second People’s Hospital of Lianyungang & The Oncology Hospital of Lianyungang, Xuzhou Medical University Lianyungang Second Hospital & Jiangsu University Lianyungang Second Hospital, Lianyungang, China; ^3^ Shanghai Tenth People’s Hospital, Tongji University School of Medicine, Shanghai, China; ^4^ Department of clinical laboratory, Obstetrics and Gynecology Hospital of Fudan University, Shanghai, China

**Keywords:** juvenile idiopathic arthritis, primary ovarian failure, Mendelian randomization, primary ovarian infertility (POI), primary ovarian failure/insufficiency

## Abstract

**Background:**

The causal relationship between juvenile idiopathic arthritis (JIA) and primary ovarian failure (POF) remains uncertain. To elucidate this relationship, we employed a two-sample Mendelian randomization analysis.

**Methods:**

The single nucleotide polymorphisms (SNPs) associated with JIA were obtained from a previously published genome-wide association study (GWAS), while the pooled data for POF originated from the FinnGen consortium. The study populations consisted exclusively of individuals of European descent. In our Mendelian randomization analysis, we performed inverse-variance weighted analysis, weighted-median analysis, weighted-mode analysis and Mendelian randomization-Egger regression analysis, supplemented by sensitivity analyses to validate the accuracy and robustness of the findings.

**Results:**

The IVW (OR = 1.23, 95% CI 1.06-1.43; P = 0.007) and weighted median (OR = 1.25, 95% CI 1.06-1.47; P = 0.009), along with sensitivity analysis validation, provide compelling evidence of a significant causal association between JIA and POF.

**Conclusion:**

The study revealed a significant causal association between genetically predicted JIA and POF, indicating that JIA significantly elevates the risk of developing POF. Therefore, it is recommended to implement screening for premature ovarian failure in women diagnosed with JIA.

## Introduction

Primary ovarian failure, a condition observed in women below the age of 40, is characterized by diminished ovarian function, reduced production of oocytes and follicles, as well as elevated levels of gonadotropins. Consequently, it leads to impaired fertility and significantly decreased synthesis of ovarian hormones. This condition arises due to either premature depletion of ovarian follicles, accelerated destruction of follicles or inadequate response to gonadotropins. The systemic decline in estrogen levels prior to natural menopause has been associated with osteoporosis, cardiovascular disease, and potentially hastened neurodegenerative aging (1). Currently, approximately 70% of primary ovarian failure can be attributed to genetic factors, including Turner syndrome (X chromosome loss) and chimerism, which are recognized as etiological factors for primary ovarian insufficiency (2, 3). Additionally, autoimmune diseases account for 10%-30% of patients with primary ovarian failure, encompassing conditions such as autoimmune thyroid disease, Addison’s disease, and type 1 diabetes (4).

Juvenile idiopathic arthritis (JIA) is the most prevalent chronic inflammatory rheumatic disease in childhood (5). It represents an autoimmune disorder, yet JIA encompasses a spectrum of arthritic conditions with unknown etiology. Typically manifesting before the age of 16, it persists for a duration exceeding six weeks (6). Among the seven types of JIA, oligoarthritis and rheumatoid factor-negative polyarthritis account for 40%-80% of cases, with a higher prevalence observed in women (5). Although autoimmune diseases such as Hashimoto’s disease, systemic lupus erythematosus, rheumatoid arthritis, psoriasis, and Crohn’s disease have been observed in patients with primary ovarian failure in current studies (7), limited research has been conducted on the association between juvenile idiopathic arthritis and primary ovarian failure. Therefore, it remains unclear whether juvenile idiopathic arthritis can contribute to the development of primary ovarian failure.

Mendelian randomization, a conventional statistical approach, employs genetic variation in risk factors as an instrumental variable to evaluate causal associations between risk factors and diseases. This method effectively mitigates potential confounding variables and reverse causality by isolating and randomly assigning samples at conception. In this study, we employed Mendelian randomization to investigate the potential association between juvenile idiopathic arthritis and primary ovarian failure (8).

## Methods

### Study design

**Figure 1:**
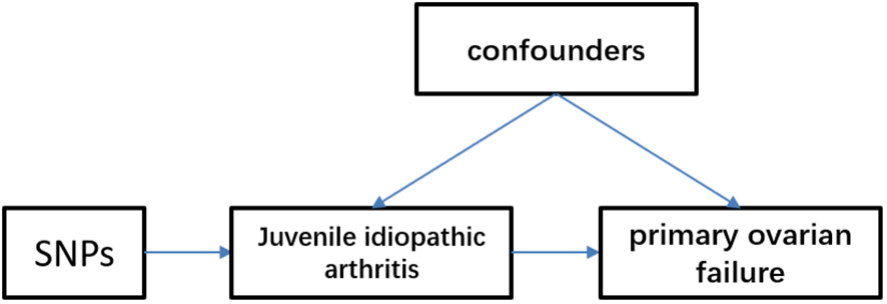
Mendelian randomization model of juvenile idiopathic arthritis and primary ovarian failure. We identified single nucleotide polymorphisms (SNPs) associated with juvenile idiopathic arthritis and estimated their corresponding effects on the risk of primary ovarian failure in a large European population cohort.

The Mendelian randomization approach utilizes publicly available datasets from genome-wide association studies (GWAS) of risk factors and diseases to investigate the causal effect of exposure on disease occurrence. In this approach, genetic variation is treated as an instrumental variable, enabling the overcoming of unmeasured confounding factors and resulting in more reliable causal inferences. The validity of Mendelian randomization relies on three assumptions: strong association between genetic variation and exposure, independence of genetic variation from other confounding factors, and genetic variation influencing outcomes solely through the investigated exposure. We obtained publicly available summaries from ethically approved published studies. No additional ethical review was necessary. To explore the causal relationship between juvenile idiopathic arthritis (JIA) and primary ovarian failure (POF), we employed a two-sample Mendelian randomization approach (**Figure 1**).

### Data sources

The data utilized in this study was obtained from the latest GWAS conducted on JIA, encompassing a European population sample of 2816 cases and 13056 controls. Within this dataset, there were a total of 103767 single nucleotide polymorphisms (SNPs) (9). The inclusion criteria of JIA came from the International League of Associations for Rheumatology (ILAR), and the included types of JIA included rheumatoid factor (RF) negative polyarticular and oligoarticular JIA (including persistent and extended JIA) (9, 10). The GWAS data for primary ovarian failure was obtained from the European FinnGen project, a multinational research initiative in Europe, accessible at https://gwas.mrcieu.ac.uk/datasets/finn-b-E4_OVARFAIL/. The dataset comprised individuals of European descent and encompassed 254 cases and 118,228 controls, encompassing a total of 16,379,677 single nucleotide polymorphisms. The inclusion criteria of POF came from International Classification of Diseases 10 (ICD-10), which was defined as estrogen deficiency, premature menopause, and resistant ovarian syndrome, excluding menopause and female climacteric status, simple gonadal dysgenesis and Turner syndrome.

According to the threshold requirement of a genome-wide significance level (P < 5 × 10^-8^) and the minimum instrumental variables criterion for Mendelian randomization studies, we selected instrumental variables with a *P* less than 5 × 10^-7^. We identified six SNPs associated with JIA for Mendelian randomization analysis, while considering parameters such as kb = 10000 and r^2^ = 0.001 to eliminate linkage disequilibrium between variables. The *F* statistic is employed to evaluate the robustness of the instrument. An *F* statistic exceeding 10 indicates that the instrument possesses sufficient strength to mitigate bias arising from weak instrumental variables (11). Exclude SNPs that exhibit a strong association with the outcome, ensuring that the *P* of both the SNP and outcome exceeds 5 × 10^-5^. Any SNP with a *P* below this threshold should be omitted from our analysis. Ultimately, only five SNPs were considered for further investigation. Details of the 5 finalized instrumental variables are listed in **Table ​1**.

**Table 1:** The detailed information of finalized single-nucleotide polymorphisms in exposure and outcomes.

SNP	EA	OA	Exposure(Juvenile idiopathic arthritis)				Outcome(Primary ovarian failure)				
			SE	Beta	P value	EAF	SE	Beta	P value	EAF	F
rs3828875	T	C	0.121	0.649	8.21E-08	NA	0.206	-0.091	0.657	0.048	28.755
rs6679677	A	C	0.075	0.567	6.64E-14	NA	0.125	0.145	0.247	0.148	56.172
rs8179673	T	C	0.059	-0.323	6.20E-08	NA	0.105	-0.203	0.055	0.768	29.299
rs9272105	A	G	0.060	0.995	6.77E-61	NA	0.092	0.217	0.018	0.605	271.029
rs9277756	A	G	0.058	0.435	1.54E-13	NA	0.113	0.003	0.979	0.189	54.518

EA, effect allele; EAF, effect allele, frequency; NA, not applicable; OA, other allele; SE, standard errors; SNP, single-nucleotide polymorphisms.

### Statistical analysis

We employed a two-sample Mendelian randomization approach to estimate the direct impact of JIA on the risk of primary ovarian failure (12). The Mendelian randomization analysis utilized an inverse-variance weighted (IVW) model, a weighted-median estimator (WME), and a Mendelian randomization-Egger regression (13–16). The IVW method was primarily employed to assess the causal effect of JIA on primary ovarian failure. Differences among various instrumental variables were assessed using Cochran’s Q test for heterogeneity (17). We employ the MR-PRESSO approach to identify and rectify the influence of potential outliers in multiple directions (18). All data analyses were performed using R (version 4.3.0) software.

## Results

### Main results

**Figure 2:**
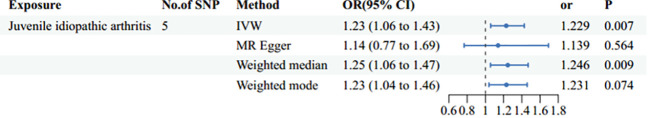
Mendelian randomization estimates of the juvenile idiopathic arthritis and risk of primary ovarian failure using different methods of Mendelian randomization: inverse variance weighted (IVW), MR-Egger, and weighted median and mode.

**Figure 3:**
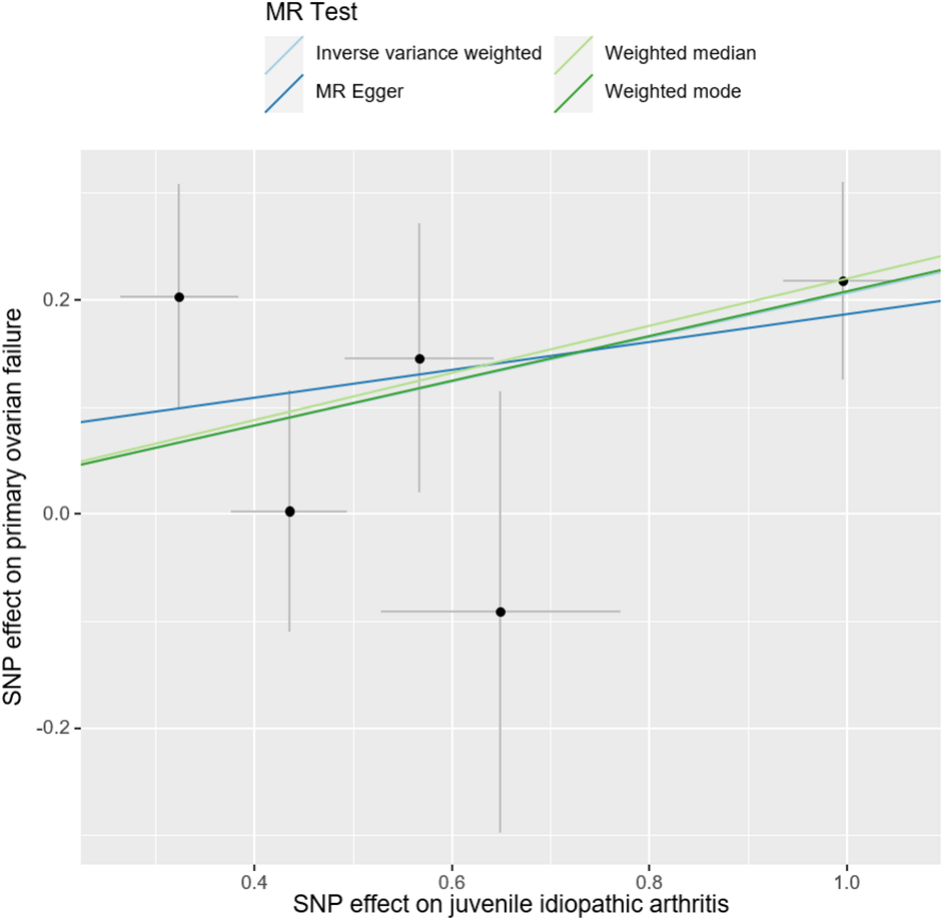
Scatter plots for Mendelian randomization analysis of juvenile idiopathic arthritis and risk of primary ovarian failure. Horizontal axis: association of SNPs with JIA. Vertical axis: association of SNPs with POF.

**Figure 4:**
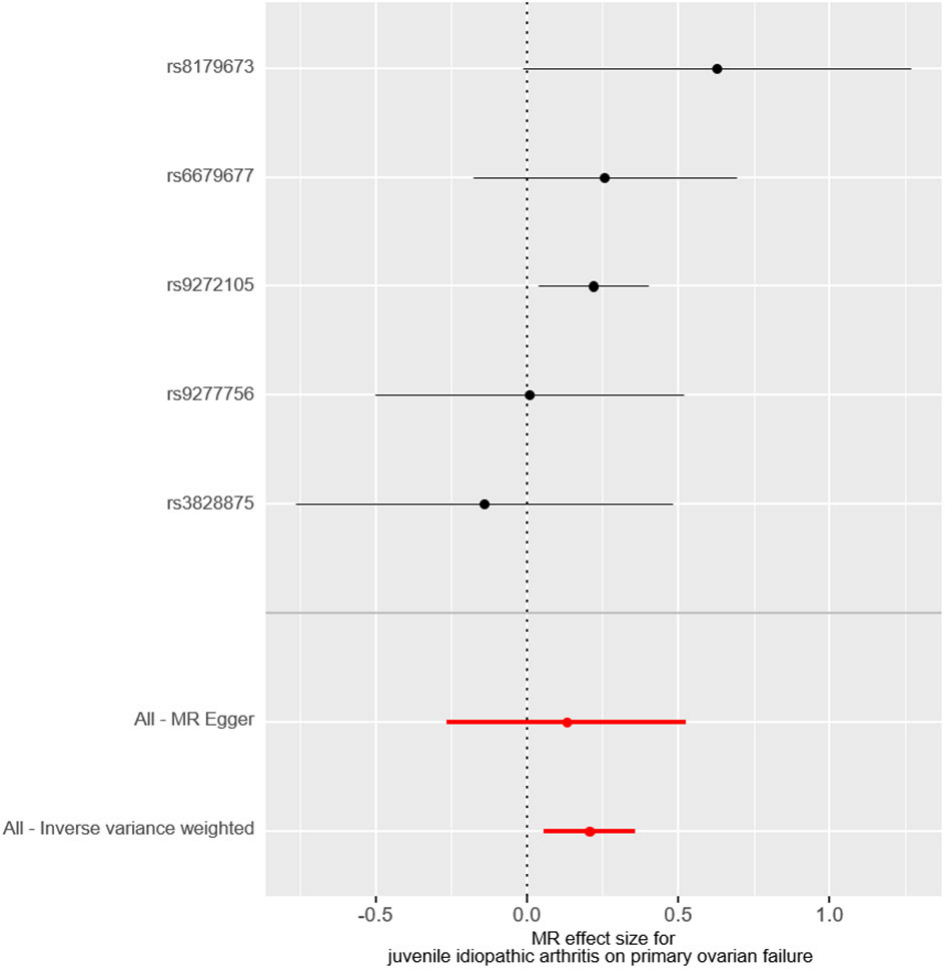
The forest plot visually demonstrates the causal effect of each single-nucleotide polymorphisms on the risk of primary ovarian failure.

The causal effect of JIA on the risk of POF was investigated using three Mendelian randomization methods, namely IVW, Mendelian randomization-Egger, and weighted median regression (**Figures 2**, **3**). The IVW method revealed a positive association between JIA and POF risk [IVW Mendelian randomization odds ratio (OR) = 1.23, 95% CI 1.06-1.43, P = 0.007]. Similar results were obtained with the weighted median method [weighted median OR = 1.25, 95% CI 1.06-1.47, P = 0.009]. No significant causal effect was observed in the MR-Egger analysis (OR = 1.14, 95% CI:0.77-1.69; P=0.564). The impact of each genetic variant on POF is illustrated in **Figures 3**, **4**.

### Sensitivity analysis

#### Analysis of heterogeneity and horizontal pleiotropy

**Figure 5:**
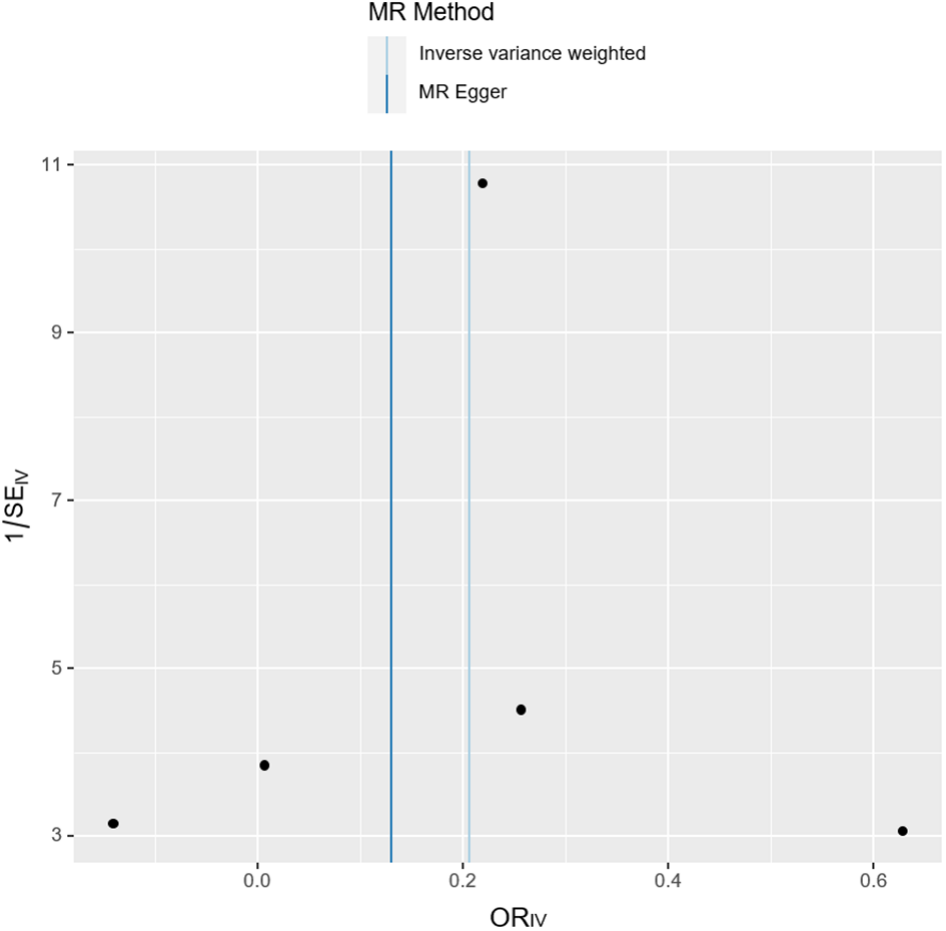
The funnel plot shows the overall heterogeneity of the Mendelian randomization estimates of the effect of juvenile idiopathic arthritis on the risk of primary ovarian failure.

The funnel plot exhibited a symmetrical comparison of individual Wald rates and their accuracy for each SNPs (**Figure 5**). Subsequently, Cochran’s Q test was conducted to assess heterogeneity among the instrumental variables, which revealed no significant heterogeneity (**Table 2**). However, due to limited interpretability of the funnel plot in evaluating horizontal pleiotropy for a few instrumental variables, additional analyses were performed using Mendelian randomization-Egger intercept and MR-PRESSO test, both of which indicated no evidence of horizontal pleiotropy (**Table 2**).

**Table 2:** Summary of heterogeneity tests and horizontal pleiotropy tests.

Method	Value	*P*
Cochran’s Q		
MR-Egger	**3.33**	**0.34**
Inverse variance weighted	**3.52**	**0.47**
MR-Egger intercept analysis	**-**	**0.71**
MR-PRESSO method	**-**	**0.65**

## Discussion

Based on the randomized Mendelian study, our research preliminarily demonstrated a significantly heightened risk of primary ovarian failure in patients with JIA. Furthermore, our sensitivity analysis confirmed the robustness of these findings, indicating that confounding factors and inverse causality did not distort the relationship between JIA and primary ovarian failure. The results obtained from IVW analysis revealed a substantial causal association between JIA and primary ovarian failure.

According to a previous observational study, primary ovarian failure unrelated to drug use was observed in 3.4% of the 187 women with long-term juvenile idiopathic arthritis (19). As ovarian reserve function decline is an early clinical manifestation of POF (4), a recent cohort study comprehensively evaluated the ovarian reserve function in post-adolescent patients with JIA. The study revealed a significant reduction in anti-Müllerian hormone (AMH) levels and the number of ovarian follicles among JIA patients, indicating a decline in ovarian reserve function among women of childbearing age. Furthermore, this decline was found to be independent of the hypothalamic-pituitary-gonadal axis (20). These studies suggest that JIA may constitute an independent risk factor for POF.

A recent study assessed ovarian reserve function in female patients with the autoimmune disease vitiligo, comparing 27 vitiligo patients with 44 healthy controls. The study found that anti-Müllerian hormone (AMH) levels and total follicle count were significantly lower in vitiligo patients compared to healthy controls, and one case of premature ovarian failure (POF) was identified in the vitiligo group, which differed significantly from the healthy controls (21). Furthermore, a recent retrospective cohort study revealed a strong association between autoimmune thyroid diseases (Hashimoto’s and Grave’s diseases) and diminished ovarian reserve as well as POF (22). Other studies have also reported a 5% occurrence of POF in women with autoimmune polyglandular syndrome-4 (APS-4) (23). Additionally, autoimmune diseases such as Addison’s disease and systemic lupus erythematosus (SLE) pose a risk for POF, with SLE carrying a risk ranging from 0.6% to 43% (24). These findings further support the impact of autoimmune processes on the ovaries of female patients with autoimmune diseases.

The mechanism by which JIA causes POF is not clear. Initially, JIA could potentially impact the onset and progression of POF via immune cell pathways. According to current research, the histopathological characteristics of JIA encompass infiltration of various immune cells, including lymphocytes, plasma cells, macrophages, and dendritic cells (25). T and B lymphocytes play a dominant role in this process, with observed activation of CD4+ and CD8+ T cells. B cells contribute by producing autoantibodies, presenting peptides and lipid antigens, as well as secreting a range of proinflammatory and anti-inflammatory cytokines (5). In autoimmune POF, monocytes and lymphocytes infiltrate developing follicles while peripheral vascular and nerve areas within the ovary are affected. Immunohistochemistry analysis reveals that T cells (CD4+ and CD8+) primarily drive cellular destruction. Additionally, B cells secrete autoantibodies (mainly IgG) which target steroid-producing cells leading to reduced numbers of ovarian follicles along with fibrosis in the ovary ultimately resulting in ovarian tissue atrophy (7, 24, 26). It can be hypothesized that JIA triggers an immune response against ovarian tissue through activation of both T and B cell populations causing infiltration by lymphocytes thereby inducing damage to the ovaries including destruction of ovarian follicles leading to POF formation. Additionally, recent studies utilizing single-cell data analysis to compare samples from normal controls and JIA have identified NK cells as the immune cells associated with JIA (27). NK cells also demonstrate enrichment of proinflammatory pathways, reduced expression of immunomodulatory genes, and impaired cytotoxic functions. Furthermore, the compromised killing activity of NK cells may contribute to extensive activation and expansion of cytotoxic CD8 T lymphocytes, production of IFNγ and other macrophage activating factors, which in turn stimulate macrophages for prolonged periods, resulting in excessive activation and expansion of macrophages that induce the onset and progression of JIA through heightened inflammatory immune activation (28). Moreover, another study observed abnormal changes in NK cells following the same single-cell data analysis of POF (29); it can be inferred that these abnormal changes in NK cells predispose individuals to long-term inflammation, potentially contributing to ovarian dysfunction and diminished ovarian reserve.

Secondly, JIA may impact the onset and progression of POF via cytokines. Proinflammatory cytokines, such as interleukin-1 (IL-1) and IL-6, are known to play a crucial role in the pathogenesis of JIA (30). Recent studies have highlighted the significance of IL-10, an anti-inflammatory cytokine, in regulating inflammation. However, it has been observed that there is a deficiency in IL-10 production in both JIA mouse models and patients, which may not be sufficient to counteract the effects of pro-inflammatory cytokines (31). Additionally, the disease activity of JIA is correlated with reduced IL-10 expression (32). This suggests that the deficiency of IL-10 could potentially contribute to the development of JIA. Interestingly, a recent Mendelian randomization analysis has revealed a causal relationship between inflammatory factors and POF, indicating that reduced levels of IL-10 are associated with an increased risk of POF (33). Therefore, it can be hypothesized that the deficiency of IL-10 observed in JIA may lead to the occurrence of POF.

Third, the Janus kinase/signal transducer and activator of transcription (JAK/STAT) signaling pathway is a crucial factor in the pathogenesis of JIA (34), and recent studies have demonstrated its essential role in primordial follicle formation (35). Impaired primordial follicle formation (PPF) can lead to the development of POF (35). However, the mechanism by which JIA mediates the occurrence of POF through the JAK/STAT signaling pathway remains unclear and requires further investigation.

One of the strengths of our study lies in the utilization of a two-sample Mendelian randomization design to evaluate the causal association between JIA and POF, thereby mitigating potential biases arising from confounding factors and reverse causation. Another advantage is that our investigation on POF was confined to the European population, which helps minimize population-specific bias. Additionally, we employed diverse MR methods to account for different assumptions regarding pleiotropy, and the consistent effect estimates obtained through these approaches provide robust evidence for causal inference.

However, our study has certain limitations. Firstly, the populations included in our study were limited to Europe, thus the generalizability of causation to other populations remains uncertain. A critical limitation of our study is the inclusion of male participants in the analysis due to the lack of available GWAS data for women with JIA. Since males do not develop POF, their inclusion potentially undermines the reliability and specificity of our findings regarding the relationship between JIA and POF. This inclusion introduces a significant methodological flaw as it does not accurately reflect the causal dynamics exclusive to females. Therefore, while our analysis suggests a possible genetic linkage, the results must be interpreted with caution, emphasizing that the demonstrated association might not accurately predict the causal relationship in females. Secondly, we selected SNPs with *P* values less than 5 × 10^-7^ as instrumental variables. The small number of SNPs poses challenges in achieving result concordance and weakens any observed associations. Additionally, the less stringent significance threshold for our chosen SNPs introduces a slight bias in the instrumental variable approach. To assess the potential impact of such bias, we calculated *F* statistics which all yielded *F* values greater than 10. Thirdly, there is a substantial overlap between the exposure and outcome data samples which may introduce bias into causal estimates. Fourthly, characteristic information about JIA such as gender was not included in the currently published JIA genomics analysis that we utilized for this study. Incorporating these factors could help control for potential confounding variables and improve accuracy of results.

We conducted a Mendelian randomization study to investigate the causal relationship between JIA and POF, which is the first of its kind. Compared to traditional observational epidemiological studies, Mendelian randomization analysis offers crucial support for establishing causation between JIA and POF by addressing unmeasured confounding factors and potential issues of inverted causality that may distort observational studies. The findings from our observational study are further substantiated by the causal estimates obtained through Mendelian randomization analysis for JIA and POF. To establish causation, we identified 5 SNPs using two GWAS datasets and employed three different models (including IVW, weighted median, and Mendelian randomization-Egger regression). Through IVW analysis, we confirmed that JIA serves as a risk factor for POF with patients having a 1.23-fold higher risk compared to those without JIA. Our results underscore the significance of early assessment of ovarian reserve function in individuals with JIA along with preventive measures against ovarian failure while providing valuable genetic insights that can enhance our understanding of the pathogenesis underlying POF associated with JIA.

Future research should strive to replicate these findings in diverse populations, as well as in female patients, to ensure their generalizability across different ethnic groups and female. Furthermore, investigating the biological mechanisms underlying the link between JIA and POF could provide valuable insights into potential therapeutic targets or preventive strategies. Longitudinal studies examining the impact of JIA management strategies on ovarian reserve and function would also be beneficial, potentially guiding clinical practice to reduce the risk of POF in patients with JIA.

## Conclusions

In conclusion, our Mendelian randomization analysis, conducted on a mixed-gender dataset, offers preliminary evidence suggesting a potential genetic link between JIA and POF. However, it is important to acknowledge that the inclusion of male participants in this analysis represents a significant methodological limitation. This limitation arises from the fact that males, who do not develop POF, were included due to data constraints. Therefore, while the analysis implies a possible association, the findings must be interpreted with caution. The results cannot definitively establish JIA as a causal factor for POF in females. Given these considerations, our study emphasizes the necessity for further research utilizing gender-specific data to accurately assess the relationship between JIA and POF. Such studies are crucial to confirm these initial findings and guide clinical screening practices for premature ovarian failure specifically in female patients with JIA. Future research should focus on validating these associations in female-only cohorts to facilitate more targeted and appropriate treatment interventions.

## Data availability statement

The original contributions presented in the study are included in the article/supplementary material. Further inquiries can be directed to the corresponding authors.

## Author contributions

YM: Writing – original draft. XS: Writing – review & editing. CY: Data curation, Writing – review & editing. AS: Methodology, Supervision, Formal analysis, Investigation, Writing – review & editing.. GW: Investigation, Supervision, Writing – review & editing. DX: Investigation, Supervision, Writing – review & editing. YX: Investigation, Supervision, Writing – review & editing.
